# Aldosterone and the mineralocorticoid receptor in renal injury: A potential therapeutic target in feline chronic kidney disease

**DOI:** 10.1111/jvp.12848

**Published:** 2020-03-03

**Authors:** Sarah Spencer, Caroline Wheeler‐Jones, Jonathan Elliott

**Affiliations:** ^1^ Comparative Biomedical Sciences The Royal Veterinary College London UK

**Keywords:** aldosterone, chronic kidney disease, feline, mineralocorticoid receptor antagonists, renin–angiotensin–aldosterone system

## Abstract

There is a growing body of experimental and clinical evidence supporting mineralocorticoid receptor (MR) activation as a powerful mediator of renal damage in laboratory animals and humans. Multiple pathophysiological mechanisms are proposed, with the strongest evidence supporting aldosterone‐induced vasculopathy, exacerbation of oxidative stress and inflammation, and increased growth factor signalling promoting fibroblast proliferation and deranged extracellular matrix homeostasis. Further involvement of the MR is supported by extensive animal model experiments where MR antagonists (such as spironolactone and eplerenone) abrogate renal injury, including ischaemia‐induced damage. Additionally, clinical trials have shown MR antagonists to be beneficial in human chronic kidney disease (CKD) in terms of reducing proteinuria and cardiovascular events, though current studies have not evaluated primary end points which allow conclusions to made about whether MR antagonists reduce mortality or slow CKD progression. Although differences between human and feline CKD exist, feline CKD shares many characteristics with human disease including tubulointerstitial fibrosis. This review evaluates the evidence for the role of the MR in renal injury and summarizes the literature concerning aldosterone in feline CKD. MR antagonists may represent a promising therapeutic strategy in feline CKD.

## 1 INTRODUCTION

Beyond its physiological role in renal sodium reabsorption and potassium excretion, there is extensive experimental evidence implicating excessive aldosterone activation of mineralocorticoid receptors (MR) in nonclassical sites, including the endothelium, vascular smooth muscle cells (VSMCs), cardiomyocytes, inflammatory cells, renal podocytes and fibroblasts, in causing tissue injury. The beneficial effect of MR antagonists (MRAs) on reducing mortality in people with heart failure is well established (Pitt et al., 1999, 2013; Zannad et al., 2011), and their prescription is included in international guidelines of heart failure treatment (Ponikowski et al., 2016). Clinical studies have also demonstrated the benefit of MRAs in people with chronic kidney disease (CKD) (Bianchi, Bigazzi, & Campese, 2006; Currie et al., 2016; Sato, Hayashi, & Saruta, 2005). Indeed, rarely has preclinical experience been translated into therapeutic use more quickly and effectively than the use of MRAs. Whilst it is true that MR activation contributes to renal damage in the context of hypertension, a blood pressure‐independent effect has been demonstrated in various models of kidney injury including subtotal nephrectomy (Ibrahim & Hostetter, 1998), ischaemia\reperfusion injury (Barrera‐Chimal et al., 2015; Mejía‐Villet et al., 2007; Ramírez et al., 2009), diabetic nephropathy (Bamberg et al., 2018), glomerulonephritis (Asai et al., 2005) and calcineurin inhibitor nephrotoxicity (Feria et al., 2003).

Chronic kidney disease is the most common cause of mortality in ageing cats (O’Neill et al., 2015) and may result in significant morbidity in affected individuals. Aetiology is usually unknown on an individual basis, but pathological characteristics, namely multifocal tubulointerstitial fibrosis and chronic mononuclear tubulointerstitial inflammation, are consistent (Chakrabarti, Syme, Brown, & Elliott, 2013; McLeland, Cianciolo, Duncan, & Quimby, 2015; Zini et al., 2014). Important differences exist between feline and human CKD, as cats exhibit a lower frequency of proteinuria and glomerulonephritis compared with humans, and different risk factors for disease development exist, with hypertension and diabetes mellitus being important in people (Jha et al., 2013), and frequent vaccination and dental disease identified in feline epidemiological studies (Finch, Syme, & Eliott, 2016; Greene et al., 2014). Tubulointerstitial fibrosis is the lesion best correlated with disease severity in both cats (Chakrabarti et al., 2013; Sawashima et al., 2000; Yabuki et al., 2010) and people (Hruby et al., 1998; Nath, 1992), however, and occurs early in feline CKD (McLeland et al., 2015). Although several clinicopathological findings, including proteinuria, anaemia and hyperphosphataemia, correlate with fibrosis severity and\or survival (Boyd, Langston, Thompson, Zivin, & Imanishi, 2008; Chakrabarti et al., 2013; Chakrabarti, Syme, & Elliott, 2012; Elliott, Rawlings, Markwell, & Barber, 2000; King, Tasker, Gunn‐Moore, Gleadhill, & Strehlau, 2007; McLeland et al., 2015; Syme et al., 2006), causal and progression factors of feline CKD remain poorly understood. Recently, renal hypoxia\ischaemia, perhaps episodic in nature, has been proposed to contribute to the initiation and progression of feline CKD (Cowgill et al., 2016; Jepson, 2016). This is supported by experimental models where renal ischaemia results in morphological changes akin to those observed in naturally occurring disease (Brown et al., 2019; Schmiedt et al., 2012, 2016). Aside from the feeding of a renal diet (Ross et al., 2006), currently no effective treatments exist which are proven to significantly slow feline CKD progression. One of the benefits of a renal diet is thought to be restriction of phosphate intake (Elliott et al., 2000; Ross, Finco, & Crowell, 1982). As such, it is important to understand factors which may be associated with disease advancement so that novel therapeutic interventions may be established.

This review provides an overview of the evidence supporting the deleterious role of aldosterone\MR activation in renal injury in laboratory animals and humans and discusses its potential relevance in the context of feline CKD.

## 2 ALDOSTERONE AND THE MR

Aldosterone is a mineralocorticoid hormone produced primarily in the zona glomerulosa of the adrenal cortex whose major physiological function is to maintain sodium and potassium homeostasis and blood pressure control. Upon binding to the MR in the epithelial cells of the renal cortical collecting tubules and collecting ducts, aldosterone stimulates a cascade of events resulting in sodium reabsorption, and thus the maintenance of intravascular volume, and potassium secretion (Ponda & Hostetter, 2006). The major secretagogues of aldosterone are increased serum potassium concentration and angiotensin II (via the angiotensin type 1 receptor) (Beuschlein, 2013). Components of the renin–angiotensin–aldosterone system (RAAS) are important on both a systemic and tissue‐specific level (Nishiyama & Kobori, 2018; Siragy & Carey, 2010), and intrarenal aldosterone may act independently of circulating aldosterone levels. In fact, in humans and laboratory species, MR blockade has been shown to be beneficial in the absence of elevated plasma aldosterone levels (Du et al., 2009; Nagase, Matsui, Shibata, Gotoda, & Fujita, 2007; Nagase et al., 2006; Pitt, Remme, Zannad, & Neaton, 2003; Pitt et al., 1999) and renal MR expression is not correlated with serum aldosterone levels in people with CKD (Quinkler et al., 2005). *CYP11B2*, the gene which codes for aldosterone synthase, is expressed in the renal cortex of normal rats and is upregulated by angiotensin II (Xue & Siragy, 2005); other extra‐adrenal sites of aldosterone synthesis include the brain, blood vessels and myocardium (MacKenzie et al., 2000; Takeda et al., 1995; White, 2003).

Aldosterone acts by genomic and nongenomic mechanisms, recently reviewed by Hermidorff, Assis, and Isoldi (2017). The relative physiological and clinical relevance of these pathways remains largely unestablished. After aldosterone binds to the cytoplasmic MR, the aldosterone‐MR complex translocates to the nucleus and modulates target gene transcription (Gumz, Popp, Wingo, & Cain, 2003; Poulsen, Limbutara, Fenton, Pisitkun, & Christensen, 2018). Serum glucocorticoid kinase‐1 (Sgk‐1) is the most important MR transcript, whose expression, amongst other effects, triggers a cascade of events in the kidney that ultimately activates the epithelial sodium channel (ENaC) and causes potassium excretion (McCormick, Bhalla, Pao, & Pearce, 2005). The MR is expressed in numerous tissues besides the kidney, including cardiomyocytes, vascular endothelial and smooth muscle cells, colonocytes and inflammatory cells (Bauersachs, Jaisser, & Toto, 2015; Bertocchio, Warnock, & Jaisser, 2011; Jaffe & Mendelsohn, 2005; Lombès et al., 1992; Nguyen Dahn Cat et al., 2010). In the rodent kidney, MRs have been detected in podocytes in vitro (Lee et al., 2009; Nagase et al., 2006; Shibata et al., 2008), mesangial cells (Lai, Chen, Hao, Lin, & Gu, 2006; Nishiyama et al., 2005) and fibroblasts (Nagai et al., 2005) in addition to tubular epithelial cells. The MR has a high affinity for glucocorticoids and epithelial MR selectivity for aldosterone is thought to be protected by 11β‐hydroxysteroid dehydrogenase type 2 (11β‐HSD2), which converts active glucocorticoids (e.g. cortisol) to MR‐inactive 11‐keto analogues (e.g. cortisone) (Odermatt & Kratschmar, 2012). Cortisol may act as an MR agonist in certain tissues or under pathological conditions, however (Mihailidou et al., 2009; Ohtake et al., 2014). 11β‐HSD2 has been detected in feline kidneys, but its localization has not been described (Schipper et al., 2004).

The rapid, nongenomic actions of aldosterone are not fully characterized but include effects on cellular calcium and sodium flux, intracellular pH, release of heat‐shock proteins and protein kinase C activation (Michea et al., 2005; Tumlin et al., 1997; Uhrenholt et al., 2004; Wehling et al., 1998). Not all rapid effects are MR‐mediated; evidence suggests that aldosterone interacts with other receptors such as the G protein‐coupled oestrogen receptor (Gros, Ding, Liu, Chorazyczewski, & Feldman, 2013) and an “unknown receptor” has also been proposed (Hermidorff et al., 2017).

## 3 MINERALOCORTICOID RECEPTOR ANTAGONISTS

Spironolactone was the first MRA to be developed, initially registered for human use in 1960 as a potassium‐sparing diuretic (Ponda & Hostetter, 2006). It also possesses significant affinity for androgen and progesterone receptors (with antagonistic and agonistic actions, respectively) (Kolkhof & Borden, 2012). The second‐generation MRA, eplerenone, was developed as a more selective MRA but has reduced potency (Shavit et al., 2012; Sica, 2005). Finerenone is a third‐generation nonsteroidal MRA with greater MR selectivity than spironolactone, greater potency than eplerenone and increased renoprotective effects (Barrera‐Chimal et al., 2016; Kolkhof, Nowack, & Eitner, 2015). Aldosterone synthase inhibitors may provide a novel method for aldosterone suppression in the future (Hargovan & Ferro, 2014). Spironolactone is the only veterinary‐licensed MRA, for the treatment of congestive heart failure caused by valvular regurgitation in dogs, either alone or as a combination product with benazepril.

## 4 ALDOSTERONE IN CKD

In the early stages of CKD, RAAS activation occurs as a compensatory response to maintain glomerular filtration rate (GFR); however, chronic activation is maladaptive and leads to progressive renal injury. Angiotensin II has historically been regarded as the major mediator of RAAS‐induced renal injury, not only through its glomerular effects but also by activating proinflammatory and profibrotic pathways (Ames, Atkins, & Pitt, 2019; Eddy, 1996; Nishiyama & Kobori, 2018). Consequently, the current standard of care for CKD treatment in human medicine involves angiotensin II inhibition with angiotensin‐converting enzyme inhibitors (ACEIs) and\or angiotensin type 1 receptor blockers (ARBs). Substantial and ever‐increasing evidence in laboratory species and humans demonstrates that aldosterone also causes direct organ damage, particularly in the heart and kidneys. Aldosterone's pathophysiological actions are similar to and overlap with those of angiotensin II (Ames et al., 2019), and the interactions between the two are complex, meaning it can be difficult to discern their individual effects (Luther et al., 2012; Virdis et al., 2002).

**Figure 1:**
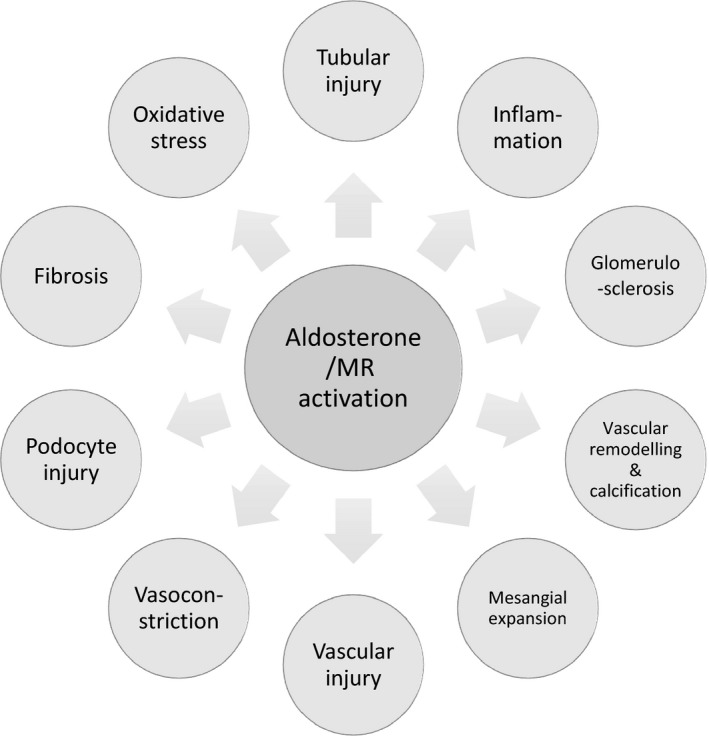
Proposed mechanisms underlying the detrimental effects of aldosterone\mineralocorticoid receptor activation on the kidney

Chronic kidney disease can be considered as a state of relative hyperaldosteronism. Increased plasma aldosterone levels are a risk factor for kidney injury in human clinical studies, and MRA treatment has been shown to be beneficial in numerous rodent models of renal disease and in human patients, for example by abrogating renal histopathological changes and reducing proteinuria and blood pressure. Aldosterone's detrimental effects on the kidney predominantly occur via nonepithelial MRs, and importantly, can arise independently of aldosterone's effect on blood pressure (Fujisawa et al., 2004; Rafiq, Hitomi, Nakano, & Nishiyama, 2010). The proposed mechanisms underlying the detrimental effects of MR activation in the kidney are outlined in Figure 1. “Aldosterone breakthrough” is a phenomenon which further supports harmful effects of MR activation; this term applies to patients on ACEI\ARB therapy who experience plasma aldosterone concentrations that return to or exceed pretreatment levels following an initial reduction (Terata et al., 2012). Aldosterone breakthrough is associated with more severe proteinuria and a faster deterioration in renal function in people (Buglioni et al., 2015; Sato, Hayashi, Naruse, & Saruta, 2003; Schjoedt, Andersen, Rossing, & Tarnow, 2004). Aldosterone breakthrough is poorly characterized in veterinary species but has been documented in cats with hypertrophic cardiomyopathy treated with ACEIs (MacDonald & Kittleson, 2008), and preliminary studies have demonstrated that aldosterone breakthrough may occur in up to 33% of dogs with proteinuric renal diseases that are receiving ACEIs\ARBs (Ames, unpublished data). Certainly, aldosterone breakthrough has been documented in dogs with cardiac disease treated with ACEIs (Ames, Atkins, Eriksson, & Hess, 2017) and in healthy dogs receiving furosemide following ACEI or ARB treatment (Ames, Atkins, Lee, Lantis, & Zumbrunnen, 2015; Konta et al., 2018; Lantis, Ames, Atkins, et al., 2015; Lantis, Ames, Werre, & Atkins, 2015).

### 4.1 Vascular effects of MR activation

The effects of MR activation on vascular function and structure is thought to be the major mechanism by which aldosterone causes renal injury (Duprez, 2007; Jaisser & Farman, 2016). MR activation in vascular endothelial cells and VSMCs results in endothelial dysfunction, increased oxidative stress (where the production of potentially damaging reactive oxygen species [ROS] exceeds endogenous antioxidant capacity) and ultimately vascular injury and remodelling, leading to reduced arterial compliance and vasoconstriction (Duprez, 2007; Gros et al., 2007; Jaffe & Mendelsohn, 2005; Nguyen Dahn Cat et al., 2010; Struthers, 2004).

#### 4.1.1 Effects on endothelial function

Endothelial dysfunction, characterized by impaired vasodilation, increased platelet and leucocyte adhesion, and decreased nitric oxide bioavailability, occurs secondary to MR activation in experimental rodent studies (Gromotowicz et al., 2011; Oberleithner et al., 2004). Aldosterone induces vascular and intercellular cell adhesion molecule (VCAM\ICAM) expression, indicating inflammatory activation of the endothelium (Lai et al., 2006), an effect reduced by MRAs (Caprio et al., 2008; Kobayashi et al., 2005). The endothelial nitric oxide synthase (eNOS)–nitric oxide pathway is key in maintaining endothelial integrity and function (Goligorsky, Brodsky, & Noiri, 2004). MR activation can reduce eNOS activity and cause eNOS uncoupling, resulting in impaired vasodilation (Arima et al., 2004; Bauersachs et al., 2015; Duprez, 2007; Gromotowicz et al., 2011; Liu, Schmuck, Chorazcyzewski, Gros, & Feldman, 2003). Oxidative stress, including enhanced ROS production, is another mechanism by which aldosterone reduces nitric oxide bioavailability and impairs vascular reactivity (Farquharson & Struthers, 2002; Leopold et al., 2007; Sanz‐Rosa et al., 2005; Virdis et al., 2002). In the kidney, impaired nitric oxide activity promotes proteinuria, accelerates innate immune system activation and causes progressive tubulointerstitial injury (Sogawa et al., 2018). eNOS uncoupling also increases hydrogen peroxide production and activates the nuclear factor‐κB pathway, leading to inflammation and fibrosis (Jaisser & Farman, 2016). Following MRA treatment, increased eNOS expression occurs and is associated with improved endothelial function and renal blood flow (Kobayashi et al., 2005; Sanz‐Rosa et al., 2005).

Circulating aldosterone levels are associated with reduced endothelial function (measured by flow‐mediated dilation) in the general population (Hannemann et al., 2011) and patients with chronic heart failure (Duprez et al., 1998), hyperaldosteronism (Nishizaka, Zaman, Green, Renfroe, & Calhoun, 2004) and low‐renin hypertension, with the latter shown to be due to impaired nitric oxide‐mediated vasodilation (Duffy et al., 2005). Endothelial dysfunction is linked to cardiovascular risk in CKD patients (Malyszko, 2010) and with prognosis in coronary heart disease (Heitzer, Schlinzig, Krohn, Meinertz, & Münzel, 2001) and hypertension (Perticone et al., 2001). People with aldosterone dysregulation show evidence of renal vascular dysfunction and have heightened cardiovascular risk (Brown et al., 2014). Improved flow‐mediated dilation with MRA treatment has been demonstrated in several conditions (Fujimura et al., 2012; Macdonald, Kennedy, & Struthers, 2004; Nishizaka et al., 2004). Plasma concentrations of asymmetric dimethylarginine (ADMA), an endogenous eNOS inhibitor, are increased in cats with CKD, suggesting that endothelial dysfunction may also occur in this species (Jepson, Syme, Vallance, & Elliott, 2008) although no direct evidence for this has been established.

#### 4.1.2 Effects on VSMCs, vascular remodelling and calcification

Endothelium\VSMC crosstalk is integral to vascular function, particularly the regulation of vascular tone. Aldosterone leads to rapid changes in calcium and sodium flux in VSMCs (Gros et al., 2007; Wehling, 2005); this mechanism has been shown to induce renal afferent and efferent arteriole vasoconstriction in rabbits, an effect not inhibited by MR blockade (Arima, Kohagura, Xu, & Sugawara, 2003). MR activation in VSMCs also leads to angiotensin II receptor upregulation (Ullian, Schelling, & Linas, 1992), inhibited nitric oxide release following cytokine stimulation (Ikeda et al., 1995) and increased expression of genes involved in vascular fibrosis, inflammation and calcification (Blasi et al., 2003; Jaffe & Mendelsohn, 2005; Virdis et al., 2002). Aldosterone is critical in renal vascular damage induced by angiotensin II and L‐NAME (an eNOS inhibitor) (Rocha et al., 2000), and also affects the plasminogen activator system, resulting in perivascular fibrosis (Aldigier, Kanjanbuch, Ma, Brown, & Fogo, 2005; Brown, Nakamura, et al., 2000), which in turn exacerbates ongoing tissue hypoxia.

Chronic MR activation results in structural vascular changes. Hypertrophic remodelling of renal small arteries occurs in aldosterone‐infused rats, an effect inhibited not only by spironolactone but also by endothelin‐1 type A (ET_A_) receptor antagonism, indicating the likely underlying mechanism (Pu, Neves, Virdis, Touyz, & Schiffrin, 2003). MR blockade improves carotid intima–media remodelling in haemodialysis patients (Vukusich et al., 2010), decreases angiotensin II‐mediated cardiac endothelial cell and VSMC hypertrophy (Hatakeyama et al., 1994), cerebral vascular remodelling in stroke‐prone rats (Rigsby, Pollock, & Dorrance, 2007) and arteriosclerosis in Dahl salt‐sensitive rats (Kobayashi et al., 2005). Vascular calcification is another feature of MR‐induced vasculopathy (Jaffe & Mendelsohn, 2005; Voelkl, Alesutan, Leibrock, Kuro‐o, & Lang, 2013); evidence suggests interplay between MR activation and the klotho fibroblast growth factor (FGF)‐23 axis, which drives soft tissue and vascular mineralization in CKD‐mineral and bone disorder (Voelkl et al., 2013; Zhang et al., 2016). Increased circulating FGF‐23 concentrations were the strongest independent predictor of feline CKD progression and all‐cause mortality in one study (Geddes, Elliott, & Syme, 2015). Although vascular calcification has not been demonstrated in cats with CKD, mineralization of other tissues occurs and serum calcification propensity (an in vitro assay which predicts vascular calcification in humans) increases with declining renal function (van den Broek, Chang, Elliott, & Jepson, 2018b). As MR activation is likely to contribute to CKD‐mineral and bone disorder in cats as in other species, further rationale exists for the use of MRAs in the management of feline CKD. MRA treatment in people with end‐stage renal disease is associated with a reduced risk of cerebro‐ and cardiovascular events (Matsumoto et al., 2014) and a reduction in vascular mineralization and stiffness likely accounts for this.

#### 4.1.3 Effects on blood pressure

Traditionally, aldosterone was believed to increase systemic blood pressure solely by sodium and volume retention. However, it is now known to act directly on the vasculature, as discussed above, and also on the central nervous system (Duprez, 2007; Shavit et al., 2012). Aldosterone potentiates vasopressor‐induced vasoconstriction in vitro (Michea et al., 2005; Nguyen Dahn Cat et al., 2010) but has little or no effect on blood pressure or systemic vascular resistance in healthy people (Farquharson & Struthers, 2002; Wehling et al., 1998); it is proposed that counteractive vasodilatory nitric oxide‐dependent pathways lost in the presence of endothelial damage attenuate aldosterone's effect on vascular tone (Arima et al., 2004; Uhrenholt et al., 2004).

Sodium and volume retention caused by MR activation contributes to renal damage (including vascular and glomerular sclerosis, tubular damage and inflammation) in rodent experimental models of hypertension (Blasi et al., 2003; Nishiyama et al., 2004; Sun et al., 2006), and protection conferred by MRA blockade can occur partly due to decreases in systolic blood pressure (Du et al., 2009; Martín‐Fernández et al., 2016; Zhou et al., 2011). Hypertension is observed in 19%–65% of cats with CKD (Acierno et al., 2018). Although hypertension has not been independently associated with CKD progression or survival (Chakrabarti et al., 2012; Jepson, Brodbelt, Vallance, Syme, & Elliott, 2009; Syme et al., 2006), it is likely that untreated hypertension results in more severe renal injury and disease progression, as in people (Jamerson & Townsend, 2011). The strong association between hypertension and proteinuria also tends to “mask” significant associations between blood pressure and CKD progression in multivariate models. MRAs are effective in reducing blood pressure in people with CKD and end‐stage renal disease (Bianchi et al., 2006; Bolignano, Palmer, Navaneethan, & Strippoli, 2014; Pisoni et al., 2012; Shavit et al., 2012), although some studies have shown no effect, likely due to differences in treatment duration and patient inclusion criteria (Chrysostomou, Pedagogos, MacGregor, & Becker, 2006; Rachmani et al., 2004; Sato et al., 2003, 2005). Hypertensive human CKD patients have more severe renal injury, lower creatinine clearance and higher serum aldosterone concentrations than their normotensive counterparts but interestingly no difference in renal MR or Sgk‐1 expression (Quinkler et al., 2005). Plasma aldosterone levels are also increased in hypertensive CKD cats when compared to normotensive cats (Jensen, Henik, & Brownfield, 1997; Jepson, Syme, & Elliott, 2014; Mishina et al., 1998). The first‐line treatment for feline hypertension is the calcium channel blocker amlodipine; although amlodipine can cause RAAS activation and aldosterone breakthrough in dogs (Ames, Atkins, Lantis, & Zum Brunnen, 2016), its effect on RAAS in cats is less clear with one study showing increased plasma renin activity but not plasma aldosterone in hypertensive cats postamlodipine treatment compared with pretreatment (Jepson et al., 2014). MRAs may have additional benefits with regard to reducing proteinuria in this population, however, as in people (White et al., 2003). Hypomagnesaemia is associated with systemic hypertension in cats with CKD (van den Broek, Chang, Elliott, & Jepson, 2018a) and MR activation may provide the link between these factors, as urinary magnesium excretion is stimulated by aldosterone (Barr et al., 1995) and aldosterone secretion is inhibited by increased circulating magnesium levels (Atarashi, Matsuoka, Takagi, & Sugimoto, 1989).

### 4.2 Ischaemic kidney injury

Renin–angiotensin–aldosterone system activation is both a potential cause and effect of renal hypoxia\ischaemia. RAAS‐driven glomerulosclerosis, haemodynamic adaptive alterations and arteriolosclerosis reduce renal capillary oxygen delivery (Hollenberg, 2004; Nangaku, 2006). Uninephrectomy plus ischaemia in rats leads to greater plasma aldosterone levels, hypertension, proteinuria and glomerulosclerosis compared with equivalent surgical reduction alone (Ibrahim & Hostetter, 1998). Sgk‐1 expression, indicating MR activation, is upregulated in vitro in human embryonic kidney cells and in vivo in mice exposed to hypoxia (Rusai et al., 2009).

Mineralocorticoid receptor activation has been investigated experimentally in renal ischaemia\reperfusion injury in rodents and the potential therapeutic use of MRAs in this setting is relevant to the hypothesis that renal ischaemia contributes to feline CKD initiation and progression (Brown et al., 2019; Cowgill et al., 2016; Jepson, 2016). Table 1 summarizes the studies investigating the effects of MR activation on renal hypoxia\ischaemia. Spironolactone prior to renal ischaemia\reperfusion protects against decreased GFR and tubular blood flow and results in reduced severity of histopathological lesions and proteinuria (Barrera‐Chimal et al., 2013, 2015; Mejía‐Villet et al., 2007; Sánchez‐Pozos et al., 2012). Protection is at least partly mediated by augmented eNOS activation (important for re‐establishing blood flow), indicated by increased urinary nitrite\nitrate ratio (Mejía‐Villet et al., 2007). MR blockade around the time of renal ischaemia is protective against progression of acute kidney injury (AKI) to CKD (Barrera‐Chimal et al., 2013, 2015, 2018; Lattenist et al., 2017). Adrenalectomy is likewise protective in these models (Ramírez et al., 2009). As well as enhanced eNOS activation, downregulation of the ET_A_ receptor (which mediates vasoconstriction) and upregulation of the endothelin type B (ET_B_) receptor (vasodilatory effect) are critical effects of MRA treatment (Barrera‐Chimal et al., 2016, 2018; Ramírez et al., 2009). Activation of the Rho\Rho‐kinase pathway, resulting in calcium‐sensitization and smooth muscle contraction, also plays a role in aldosterone's vasoconstrictive and profibrotic effects following renal ischaemia (Kobayashi et al., 2005; Ramírez et al., 2009; Sun et al., 2006). MRAs likewise provide protection against ischaemic injury in other tissues (Fujita et al., 2005; Oyamada et al., 2008; Ozacmak, Ozacmak, Barut, Arasli, & Ucan, 2014).

**Table 1:** Studies investigating the effects of aldosterone\MR activation on renal hypoxia\ischaemia

Reference	Species	Model\population	Results
RI studies			
Barrera‐Chimal et al. (2013)	Rat	45 min of bilateral RI; spironolactone administered 3 days, 0, 1.5 or 3 hr subsequent to RI	*Spironolactone at all time points prevented CKD development:* Inhibition of activation of fibrotic and inflammatory pathways (TGFß‐1, TNF‐α, MCP‐1, IL‐6) Abrogated structural tubular and glomerular changes Prevented progressive increase in proteinuria
Barrera‐Chimal et al. (2015)	Rat	10, 20 or 45 min of bilateral RI; spironolactone administered at 0 or 1.5 hr after RI	*Spironolactone:* Prevented renal hypertrophy and tubulointerstitial fibrosis seen after 20 and 45 min of RI Prevented activation of TGF‐β signalling pathway and upregulation of ET_A_ receptor, reduced α‐SMA expression
Barrera‐Chimal et al. (2016)	Rat	25 min of bilateral RI; nonsteroidal MR antagonist BR−4628 administered 48, 24 and 1 hr before or 3 hr after RI	*BR−4628 administration at all time points:* Protected against renal dysfunction, tubular injury and oxidative stress Prevented ET_B_ receptor downregulation and decreased eNOS activation
Lattenist et al. (2017)	Rat	Acute: 25 min of bilateral RI; three doses of finerenone treatment 48, 24 and 1 hr before Chronic: 45 min of bilateral RI; finerenone treatment 1 and 2 days and 1 hr before	*Finerenone:* Acute model: prevented kidney dysfunction and tubular injury, decreased KIM−1 and NGAL expression Chronic model: prevented AKI‐to‐CKD transition, including reduced TGF‐β and collagen I expression, decreased proteinuria and renal vascular resistance
Mejía‐Villet et al. (2007)	Rat	20 min of bilateral RI; spironolactone administered 1, 2 or 3 days before RI	*Spironolactone:* Prevented decreased renal blood flow Prevented acute renal failure Prevented tubular apoptosis Decreased oxidative stress Upregulated eNOS expression, increased activating phosphorylation\decreased inactivating phosphorylation
Ramírez et al. (2009)	Rat	20 min of bilateral RI; adrenalectomy 3 days prior	*Adrenalectomized rats showed:* Prevention of decreased GFR Prevention of increased markers of oxidative stress and tubular injury Increased eNOS expression and activating phosphorylation Normalization of Rho‐kinase expression Normalization of ET_A_ receptor expression
Sánchez‐Pozos et al. (2012)	Rat	20 min of bilateral RI; spironolactone administered 0, 3, 6 and 9 hr subsequently	*Spironolactone at 0 and 3 hr after RI:* Prevented decreases in RBF and GFR Prevented tubular injury and increase in KIM−1, heat‐shock protein 72 and proteinuria Inhibited ET_A_ receptor increase and ET_B_ receptor decrease
CIN studies			
Amador et al. (2016)	Mouse	CsA treatment; targeted deletion of MR in endothelial cells or VSMCs	*MR deletion in VSMCs abrogated:* Increased renal vascular resistance Phosphorylation of contractile proteins Increase in serum creatinine NGAL overexpression
Feria et al. (2003)	Rat	21 days of CsA treatment ± spironolactone; low sodium diet	*Spironolactone:* Decreased arteriolopathy Decreased tubulointerstitial fibrosis, TGF‐β, collagen I and fibronectin expression Prevented reduced creatinine clearance
Pérez‐Rojas et al. (2005)	Rat	Acute CIN: 7 days of CsA treatment, causing 50% reduction in RBF Chronic CIN: 21 days of CsA treatment	*Spironolactone:* Acute model: prevented decreased RBF and GFR Chronic model: prevented pro‐renin upregulation, angiotensin−2 receptor increase and ET_B_ receptor downregulation
Other studies			
Arima et al. (2003)	Rabbit (in vitro)	Aldosterone added to microperfused renal afferent and efferent arterioles	Aldosterone caused dose‐dependent constriction in afferent and efferent arterioles, with a higher sensitivity in the latter. Pretreatment with neomycin (phospholipase C inhibitor) abolished vasoconstriction No effect of spironolactone (suspected nongenomic effects)
Arima et al. (2004)	Rabbit (in vitro)	Aldosterone added to microperfused renal afferent and efferent arterioles	Aldosterone caused dose‐dependent constriction in afferent and efferent arterioles. NO‐mediated in the afferent arteriole, via IP_3_ and PKC pathways
Uhrenholt et al. (2004)	Rabbit (in vitro)	Aldosterone added to renal afferent arterioles	Aldosterone inhibits depolarization‐induced vasoconstriction; effect abolished by eNOS blockade, spironolactone and PI3‐kinase inhibition
Du et al. (2009)	Rat (DS)	High‐salt diet; eplerenone, amlodipine or both administered	Amlodipine but not eplerenone ameliorated renal hypoxia, estimated by pimonidazole, VEGF expression and peritubular endothelial cell density Eplerenone attenuated glomerulosclerosis and development of proteinuria; minimal effect on interstitial fibrosis
Waanders et al. (2009)	Rat	Renal transplant model; spironolactone administered from 2 days prior	*Spironolactone:* Ameliorated transplant vasculopathy Reduced glomerular macrophage influx Trend towards reduced proteinuria and glomerulosclerosis No effect on interstitial fibrosis
Laursen et al. (2018)	Mouse	*Nr3c2* knockout (deletion of endothelial cell MR)	No effect on renal artery and afferent arteriole contraction or dilation at baseline or after AngII infusion. No effect on proteinuria or renal histology
Ojeda‐Cervantes et al. (2013)	Human	Adult renal transplant recipients; double‐blind, randomized, placebo‐controlled pilot study. Spironolactone administered 1 day before and 3 days post‐transplantation	*Spironolactone:* Reduced oxidative stress, as assessed by urinary H_2_O_2_ excretion No difference in renal function or tubular injury biomarkers
Schmidt et al. (2006)	Human	Aldosterone infusion ± l‐NMMA (eNOS inhibitor); randomized, double‐blinded fourfold crossover design in healthy men	Aldosterone alone did not affect RBF or GFR Aldosterone with l‐NMMA increased renal vascular resistance more than l‐NMMA alone, indicating aldosterone's effects are dependent on the presence of endothelial dysfunction

Abbreviations: CIN, cyclosporine‐induced nephropathy; CsA, cyclosporine‐A; eNOS, endothelial nitric oxide synthase; ET, endothelin; GFR, glomerular filtration rate; H_2_O_2,_ hydrogen peroxide; IL‐6, interleukin‐6; IP_3,_ Inositol trisphosphate; KIM‐1, kidney injury molecule‐1; l‐NMMA, *N*(G) monomethyl‐l‐arginine; MCP‐1, macrophage chemoattractant protein‐1; NGAL, neutrophil gelatinase‐associated lipocalin; NO, nitric oxide; PKC, protein kinase C; RBF, renal blood flow; RI, renal ischaemia\reperfusion injury; TGF‐ß, transforming growth factor‐ß; TNF‐α, tissue necrosis factor‐α; VSMCs, vascular smooth muscle cells**;** α‐SMA, α‐smooth muscle actin.

Further evidence that aldosterone modulates renal ischaemia is provided by rodent experiments investigating transplant nephropathy and cyclosporine‐induced nephropathy. Vasoconstriction and altered renal haemodynamics occur in acute cyclosporine‐induced nephropathy (Amador et al., 2016; Bobadilla & Gamba, 2007) and are prevented by MR blockade (Bobadilla & Gamba, 2007; Nielsen, Jensen, Hansen, Marcussen, & Bie, 2013; Pérez‐Rojas et al., 2005), seemingly through VSMC MR inactivation (Amador et al., 2016). MRAs improve transplant‐associated vasculopathy and glomerular macrophage influx (Waanders et al., 2009), protect against chronic changes induced by cyclosporine including vasoconstriction, arteriolopathy and tubulointerstitial fibrosis (Feria et al., 2003; Nielsen, Jensen, Marcussen, Skøtt, & Bie, 2008), and slow kidney damage progression in established injury (Pérez‐Rojas et al., 2007). Experimental evidence is supported by clinical data; spironolactone reduced proteinuria post‐transplantation in human patients already receiving an ACEI and ARB (Gonzales Monte et al., 2010) and reduced markers of oxidative stress (Ojeda‐Cervantes et al., 2013). Clinical trials are ongoing to further characterize the effects of MRAs in renal transplantation (NCT01602861, NCT02490904).

Aldosterone can also contribute to renal ischaemia by promoting microthrombi in injured or dysfunctional vessels (Brown, Kim, et al., 2000; Gromotowicz et al., 2011; Rocha et al., 2000), a process mediated by oxidative stress (Stier, 2000). MRA treatment can reduce thrombosis (Rigsby et al., 2007). Lastly, MR activation may have a deleterious effect on angiogenesis (Kobayashi, Fukushima, Takeshima, Koguchi, et al., 2010; Zheng et al., 2019), although not all studies have demonstrated benefit of MRA treatment in this context (Du et al., 2009).

### 4.3 Proteinuria\glomerular damage

An enhanced MR effector mechanism is closely related to proteinuria, a strong risk factor for CKD progression in people (Heerspink, Kröpelin, Hoekman, & de Zeeuw, 2015) and prognosis in cats (Chakrabarti et al., 2012; King et al., 2007; Kuwahara, Ohba, & Kitoh, 2006; Syme et al., 2006). MR‐related proteinuria was historically considered to occur secondary to hypertension, but blood pressure‐independent effects have been demonstrated in various rodent models (Aldigier et al., 2005; Blasi et al., 2003; Brown, Nakamura, et al., 2000; Kobayashi, Fukushima, Takeshima, & Ishimitsu, 2010; Nishiyama et al., 2004; Zhou et al., 2011) and in human renal disease (Bertocchio et al., 2011; Bianchi et al., 2006; Chrysostomou et al., 2006; Sato et al., 2003, 2005; White et al., 2003). For example, eplerenone prevented renal failure, proteinuria and histological lesions in rats despite persistence of severe hypertension (Kobayashi et al., 2005). MR activation induces podocyte apoptosis and injury (Lee et al., 2009), mesangial matrix expansion (Nishiyama et al., 2005), and increases mesangial cell production of ROS, transforming growth factor (TGF)‐β1, ICAM‐1 and fibronectin (Kitada et al., 2012; Lai et al., 2006; Nagase et al., 2007; Terada et al., 2012). MRAs attenuate these effects in various rodent models of renal injury, resulting in decreased glomerulosclerosis and proteinuria (Du et al., 2009; Kobayashi et al., 2005; Luther et al., 2012; Nagase et al., 2006; Rocha et al., 2000). In the renal mass reduction model, spironolactone even led to regression of sclerotic lesions in one‐third of rats, although other groups have not corroborated this result (Aldigier et al., 2005).

In humans, plasma aldosterone levels are positively correlated with proteinuria severity in primary hyperaldosteronism (Catena et al., 2007), CKD (Bianchi et al., 2006; Bomback, Kshirsagar, Amamoo, & Klemmer, 2008) and diabetic nephropathy (Schjoedt et al., 2004). Proteinuria is correlated with MR and Sgk‐1 expression in CKD (Quinkler et al., 2005). Reduction in proteinuria is the main benefit of MRA therapy in human renal disease; numerous small randomized controlled trials have demonstrated this effect (Ando et al., 2014; Bianchi et al., 2006; Chrysostomou et al., 2006; Epstein et al., 2006; Esteghamati et al., 2013; Furumatsu et al., 2008; Gonzales Monte et al., 2010; Guney et al., 2009; Rachmani et al., 2004; Schjoedt et al., 2004; Tylicki et al., 2008), and a Cochrane review concluded that MRA treatment in addition to standard therapy is beneficial in reducing proteinuria (Bolignano et al., 2014). MRA combination therapy with an ACEI was more effective in reducing proteinuria than either drug alone (Rachmani et al., 2004), whereas triple therapy (MRA, ACEI and ARB) was no more effective than ACEI and spironolactone co‐therapy (Chrysostomou et al., 2006). Table 2 summarizes the studies investigating MR blockade on glomerular damage and proteinuria.

**Table 2:** Studies investigating the effects of MR antagonism on proteinuria and glomerular damage

Reference	Model\species\ population	Mineralocorticoid antagonist investigated	Results
Preclinical studies			
Aldigier et al. (2005)	Rats 5\6 nephrectomy	Spironolactone	84% increase in GS index (compared with 157% in controls), GS regression in some rats BP increased despite spironolactone; effects on GS were enhanced when BP was controlled by antihypertensives
Asai et al. (2005)	Rat model of glomerulonephritis	Spironolactone, also looked at the effect of cilazapril (ACEI)	Reduced proteinuria (to the same degree as cilazapril)
Bamberg et al. (2018)	Uninephrectomized *db\db* mice (diabetes model) and uninephrectomized rats administered aldosterone and high salt	AZD9977 and eplerenone	Reduced UACR and GS
Blasi et al. (2003)	Uninephrectomized rats, aldosterone\salt treatment	Eplerenone	Reduced albuminuria and glomerular injury lesions
Brown, Nakamura, et al. (2000)	Rats with radiation injury	Spironolactone, also looked at an AngI antagonist	Reduced proteinuria and GS (BP‐independent effects) Combination therapy had a greater effect on proteinuria than spironolactone alone
Du et al. (2009)	DS rats	Eplerenone, also looked at the effect of amlodipine	Reduced proteinuria and BP Superior to amlodipine in inhibiting GS but inferior in inhibiting tubulointerstitial fibrosis
Gullulu, Akdag, Kahvecioglu, Filiz, and Savci (2006)	Rat model of glomerulonephritis	Spironolactone, also looked at the effect of valsartan (ARB)	Reduced GS and TGF‐β1 expression
Guo et al. (2006)	Uninephrectomized type 1 (streptozotocin‐treated rat) and type 2 (*db\db* mouse) diabetes models	Eplerenone	Reduced albuminuria, podocyte injury, fibrosis, glomerular hypertrophy and mesangial expansion (BP‐independent effects)
Huang et al. (2012)	Mouse, unilateral ureteral obstruction	Eplerenone	Reduced albuminuria, GS and glomerular crescents, infiltration of inflammatory cells, proinflammatory cytokines Podocyte‐specific MR deletion had no effect
Kang et al. (2009)	Diabetic rats Cultured mesangial cells treated with high glucose and aldosterone	Eplerenone, also looked at the effect of enalaprilat (ACEI)	Dose‐dependent reduction in albuminuria and GS Decreased expression of TGF‐β1, type IV collagen and PAI‐1 Synergistic effect with enalaprilat
Kobayashi et al. (2005)	Rats	Eplerenone	Prevented renal failure, proteinuria and histological lesions despite persistence of severe hypertension
Kobayashi et al. (2005)	Salt‐treated DS rats	Eplerenone	Decreased GS and proteinuria
Lee et al. (2009)	Podocytes in vitro under diabetic conditions Rats with streptozotocin‐induced diabetes	Spironolactone	Inhibited podocyte apoptosis and injury
Luther et al. (2012)	Aldosterone synthase knockout mice and wild‐type littermates, treated with AngII or vehicle plus salt loading	Spironolactone	Reduced glomerular hypertrophy (aldosterone deficiency did not) AngII\salt promoted glomerular injury via the MR in aldosterone synthase knockout mice
Nagase et al. (2006) and Nagase et al. (2007)	Rat model of metabolic syndrome	Eplerenone, plus looked at effect of tempol (antioxidant)	Reduced podocyte injury (evidenced by foot process effacement, induction of desmin and attenuation of nephrin) Delayed progression of proteinuria and GS, as did tempol
Nishiyama et al. (2004)	Rats, aldosterone\salt treatment	Eplerenone, also looked at effect of tempol (antioxidant)	Reduced proteinuria, as did tempol
Nishiyama et al. (2005)	Cultured rat mesangial cells	Eplerenone	Attenuated aldosterone‐induced ERK1\2 phosphorylation Prevented the cellular proliferative and deforming effects of aldosterone
Nishiyama et al. (2010)	Diabetic rats	Eplerenone, also looked at the effect of telmisartan (ARB)	Decreased proteinuria, GS and podocyte injury Synergistic effect with telmisartan
Rocha et al. (2000)	AngII and L‐NAME treated (nitric oxide synthase inhibitor) and salt‐loaded rats	Adrenalectomy or eplerenone	Abrogated proteinuria; aldosterone administration to adrenalectomized rats restored proteinuria
Shibata et al. (2008)	Mice with increased Rac1 activity	Eplerenone	Prevented albuminuria and podocyte injury
Terada et al. (2005)	Rat cultured mesangial cells and rat isolated glomeruli	Spironolactone	Aldosterone stimulated mesangial cell proliferation by activating mitogen‐activated protein kinase 1\2, cyclin D1 and cyclin A pathways; spironolactone inhibited these effects
Zhou et al. (2011)	DS rats fed high‐salt diet	Eplerenone	Reduced proteinuria and glomerular injury score
Clinical studies			
Ando et al. (2014)	RCT, hypertensive patients with nondiabetic CKD	Eplerenone (in addition to ACE and\or ARB)	Reduced UACR
Bakris et al. (2015) (ARTS‐DN)	RCT, normotensive DN patients with high albuminuria	Finerenone (in addition to ACEI or ARB)	Dose‐dependent reduction in UACR at 90 days (study end (BP‐independent))
Bianchi et al. (2006)	Randomized open‐label study; patients with CKD (non‐DN)	Spironolactone (in addition to ACEI and\or ARB)	Additional antiproteinuric effect Baseline aldosterone levels were correlated with proteinuria and predicted degree of proteinuria reduction with spironolactone
Bolignano et al. (2014)	Meta‐analysis (2002–2011); 1549 CKD patients (nondialysis)	Spironolactone and eplerenone (in addition to an ACEI and\or ARB)	Concluded that MRAs effectively reduce proteinuria when used in combination with ACEIs and ARBs No effect on short‐term eGFR
Chrysostomou et al. (2006)	RCT, CKD	Spironolactone (in addition to ACE ± ARB)	Greater reduction in protein excretion occurred in treatment regimens that incorporated spironolactone, sustained at 6 and 12 months No advantage of triple blockade over dual RAS blockade
Currie et al. (2016)	Meta‐analysis (2005–2014); 1,646 CKD patients (nondialysis)	Spironolactone and eplerenone (in addition to an ACEI or ARB or both)	Reduced weighted mean protein\albumin excretion by 38.7% Slightly deleterious short‐term impact on eGFR
Epstein et al. (2006)	DN	Eplerenone (in addition to ACEI)	Reduced UACR compared with placebo; comparable between 50 mg and 100 mg dosages
Esteghamati et al. (2013)	RCT DN	Spironolactone\ARB vs. ACEI\ARB	Greater reduction in proteinuria after 18 months, independent of BP (decreased urinary albumin excretion by 46, 72 and 59% after 3, 12 and 18 months) No difference in eGFR decline rate between groups
Furumatsu et al. (2008)	RCT, patients with nondiabetic CKD	Spironolactone (in addition to ACEI and ARB)	Reduced proteinuria compared with baseline by 58%, no change in controls Reduced urinary type IV collagen level
Gonzales Monte et al. (2010)	Kidney transplant recipients with severe proteinuria	Spironolactone (in addition to ARB and ACEI)	>50% reduction in proteinuria in 9\11 patients, sustained at 6 months
Guney et al. (2009)	Nondiabetic CKD	Spironolactone (in addition to ACE and\or ARB)	Reduction in UPCR at 6 months Reduction in urinary TGF‐β1 excretion
Hou, Xiong, Cao, Wen, and Li (2015)	Meta‐analysis of patients with DN	Spironolactone (in addition to ACEI or ARB)	Reduced 24‐hr urinary albumin\protein excretion and UACR Significantly reduced BP was also reported, therefore proteinuria reduction may have been partly due to BP‐lowering effects
Pitt et al. (2013) (ARTS)	RCT, open‐label; heart failure patients with mild or moderate CKD	Finerenone vs. spironolactone	Finerenone was equivalent to spironolactone in decreasing albuminuria Finerenone was associated with a lower incidence of hyperkalaemia and worsening renal function
Rachmani et al. (2004)	Patients with DN and hypertension	Spironolactone, cilazapril or their combination	Spironolactone was superior to cilazapril in reducing UACR Co‐therapy more effective than either drug alone BP‐independent effects
Sato et al. (2003)	Patients with DN	Spironolactone (in addition to ACEI)	Reduced urinary albumin excretion by 40% Effect higher in patients with aldosterone breakthrough BP independent
Sato et al. (2005)	CKD (DN and non‐DN, BP controlled)	Spironolactone (in addition to ACEI)	Reduced urinary albumin excretion, effect greater in diabetic vs. nondiabetic patients (46% vs. 29%) Reduced urinary collagen type IV
Tylicki et al. (2008)	Randomized open crossover study; nondiabetic CKD	Spironolactone (in addition to ACEI and ARB)	Triple therapy reduced 24‐hr urine protein excretion compared with dual therapy
White et al. (2003)	Patients ≥ 50 years old with systolic hypertension and widened pulse pressure; double‐blind titration to effect design	Eplerenone (vs. amlodipine)	Eplerenone more effective than amlodipine in reducing UACR (52% vs. 10%) at 24 weeks Equivalent effects on systolic BP, pulse pressure and pulse wave velocity

Abbreviations: ACEI, angiotensin‐converting enzyme inhibitor; AngII, angiotensin II; ARB, angiotensin receptor blocker; BP, blood pressure; CKD, chronic kidney disease; DN, diabetic nephropathy; DS, Dahl salt‐sensitive; eGFR, estimated glomerular filtration rate; ERK, extracellular signal‐regulated kinase; GS, glomerulosclerosis; L‐NAME, *N*(gamma)‐nitro‐L‐arginine methyl ester; MRA, mineralocorticoid receptor antagonist; PAI‐1, plasminogen activator inhibitor‐1; RAS, renin–angiotensin system; RCT, randomized controlled trial; TGF‐ß1, transforming growth factor‐ß1; UACR, urinary albumin\creatinine ratio.

### 4.4 Oxidative stress

**Figure 2:**
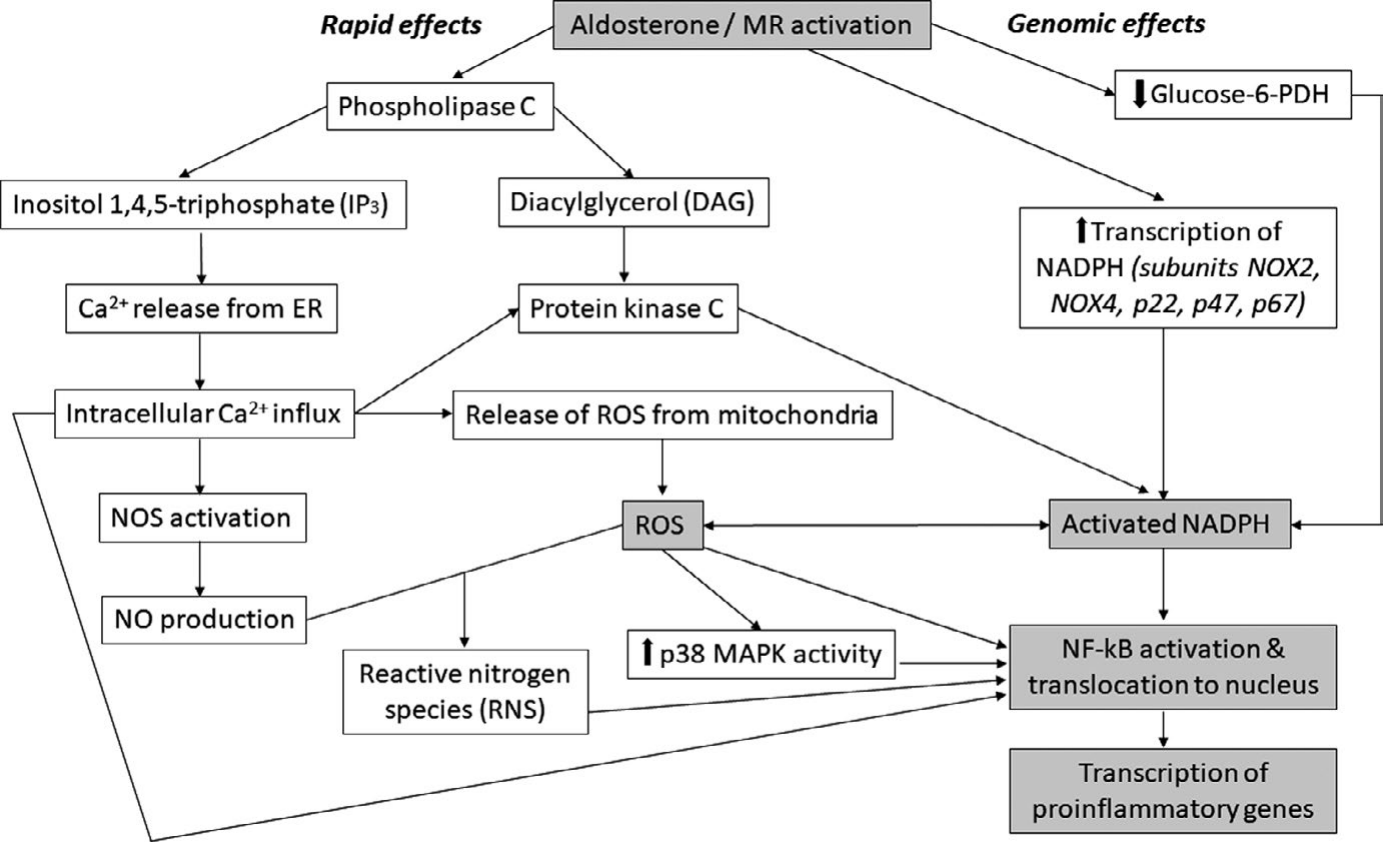
Proposed pathways responsible for the effect of mineralocorticoid receptor activation on oxidative stress. ER, endoplasmic reticulum; glucose‐6‐PDH, glucose‐6‐phosphate dehydrogenase; MAPK, mitogen‐activated protein kinase; NADPH, nicotinamide‐adenine dinucleotide phosphate; NF‐κB, nuclear factor‐ κB; NO, nitric oxide; NOS, nitric oxide synthase; ROS, reactive oxygen species

Data suggest that oxidative stress is a central mechanism by which aldosterone\MR activation causes renal damage (Nishiyama & Abe, 2006; Nishiyama et al., 2004), particularly vascular injury\endothelial dysfunction, renal cell apoptosis, inflammation and fibrosis (Leopold et al., 2007; Sanz‐Rosa et al., 2005; Sun et al., 2002; Sun, Zhang, Zhang, & Ramires, 2000; Terada et al., 2005). Aldosterone‐induced oxidative and nitrosative stress has been demonstrated in multiple cell types, including VSMCs (Maron et al., 2009), endothelial cells (Nagata et al., 2006), mesangial cells (Leopold et al., 2007), proximal tubular epithelial cells (Schupp et al., 2010) and distal tubular cells (Queisser et al., 2013), and increased urinary markers of oxidative stress are detected following MR‐induced injury in rats (Nagase et al., 2006). The pathways by which MR activation may result in oxidative stress are outlined in Figure 2. In laboratory species, MRA treatment reduces oxidative stress markers and ROS generation, and increases antioxidant enzyme mRNA expression (Mejía‐Villet et al., 2007; Queisser et al., 2013; Toyonaga et al., 2011). Further evidence is provided by the demonstration that many renal effects of MR blockade are reproduced by antioxidant treatment (Kitada et al., 2012; Nagase et al., 2006; Son et al., 2008). Reduced oxidative stress is observed in diabetic nephropathy and kidney transplant patients treated with an MRA (Ojeda‐Cervantes et al., 2013; Takebayashi, Matsumoto, Aso, & Inukai, 2006).

### 4.5 Renal inflammation and fibrosis

Renal injury induced by aldosterone\MR activation is characterized by heightened inflammation and fibrosis, and MRAs abrogate these changes in both preclinical and clinical studies. Whether aldosterone directly contributes to inflammation and fibrosis or whether these occur predominantly secondary to vascular injury is somewhat uncertain, although some experimental data suggest the latter (Rocha et al., 2000). Aldosterone\MR activation in rodents induces the renal expression of profibrotic molecules, including connective tissue growth factor (Gumz et al., 2003; Kadoya et al., 2015; Martín‐Fernández et al., 2016), plasminogen activator inhibitor‐1 (Brown, Nakamura, et al., 2000), epidermal growth factor and its receptor (Krug et al., 2003; Sheng et al., 2016), matrix metalloproteinase‐2 (Martín‐Fernández et al., 2016) and TGF‐β1 (Fujisawa et al., 2004; Kadoya et al., 2015; Lai et al., 2006; Sun et al., 2006) with successful inhibition by MRA documented in most cases. Aldosterone induces collagen synthesis in cultured fibroblasts (Nagai et al., 2005; Zhou, Kandala, Tyagi, Katwa, & Weber, 1996) and glomerular mesangial cells (Diah et al., 2008), and fibronectin synthesis (Chen et al., 2013) and osteopontin expression in renal fibroblasts (Irita et al., 2008). Aldosterone causes fibroblast proliferation due to rapid activation of growth factor receptors and induction of phosphoinositide 3‐kinase\mitogen‐activated protein kinase signalling (Huang, Nikolic‐Paterson, Ma, & Tesch, 2012). Collagen deposition is inhibited by spironolactone in vivo (Fujisawa et al., 2004). Aldosterone may also contribute to fibrosis by inducing epithelial‐to‐mesenchymal transition, seemingly via a ROS‐mediated pathway (Zhang, Jia, Guo, & Yang, 2007).

Aldosterone has been used to induce renal inflammation in rodents (Irita et al., 2011; Sogawa et al., 2018; Sun et al., 2006); leucocyte infiltration is associated with ROS accumulation and nuclear factor‐κB activation in this model (Irita et al., 2011; Queisser et al., 2013; Shibata, Nagase, Yoshida, Kawachi, & Fujita, 2007; Terada et al., 2005). Aldosterone‐infused rats show increased renal expression of proinflammatory cytokines, an effect attenuated by MRAs and MR deletion in macrophages (Blasi et al., 2003; Irita et al., 2011; Kadoya et al., 2015; Martín‐Fernández et al., 2016; Sun et al., 2006). Indeed, macrophages are key in mediating MR‐induced injury; MR activation causes macrophage polarization towards the proinflammatory M1 phenotype (Bene, Alcaide, Wortis, & Jaffe, 2014; Martín‐Fernández et al., 2016). Aldosterone\salt treatment causes perivascular leucocyte infiltration and increased expression of monocyte chemoattractant protein (MCP)‐1, interleukin (IL)‐6 and IL‐1β in the rat kidney, with MRAs being protective against this proinflammatory state (Blasi et al., 2003).

In people with CKD, renal MR and Sgk‐1 expression are positively correlated with TGF‐β1 and MCP‐1 expression, and serum aldosterone levels with renal fibrosis (Quinkler et al., 2005). Spironolactone reduces urinary TGF‐β1 levels and markers of fibrosis and tubular injury in renal biopsies in this population (Guney et al., 2009; Tylicki et al., 2008) and also urinary type IV collagen in patients with diabetic (Sato et al., 2005) and nondiabetic nephropathy (Furumatsu et al., 2008). A tendency for reduced tubulointerstitial fibrosis was also demonstrated in a small study of paediatric patients with chronic allograft nephropathy receiving eplerenone (Medeiros et al., 2017). Given that the dominant histopathological features of feline CKD are tubulointerstitial fibrosis and inflammation (Chakrabarti et al., 2013), it is proposed that MR blockade in this species would be beneficial in reducing these lesions and resultant disease progression.

## 5 FURTHER COMMENTS ON MRAS IN HUMAN CKD AND END‐STAGE RENAL DISEASE

It is important to note that although numerous studies have investigated the effect of MRAs in human CKD patients, most have focused on the reduction in proteinuria and hypertension. To date, no studies have evaluated primary end points which allow conclusions to be made about whether MRAs reduce mortality or slow CKD progression. Two small studies have suggested the latter, however, based on a slower decline in estimated GFR (eGFR) compared with control groups (Bianchi et al., 2006; Tylicki et al., 2008). Enrolment is ongoing for a trial designed to evaluate the effect of finerenone on disease progression in patients with diabetic nephropathy (NCT02540993). Additionally, studies investigating MRA treatment in severe CKD are still limited, although a meta‐analysis of dialysis patients found a reduction in mortality with the addition of MRA treatment (Quach et al., 2016). This is proposed to be due to improved cardiac function and reduced cardiovascular events. Table 3 summaries the studies investigating MRAs in the context of cardiovascular outcomes in renal disease.

**Table 3:** Studies investigating the effects of MR antagonism on cardiovascular outcomes in renal disease

Reference	Model\species\population	Mineralocorticoid antagonist investigated	Results
Preclinical studies			
Bonnard et al. (2018)	Subtotal nephrectomy CKD model in mice	Finerenone	Prevented cardiac diastolic dysfunction, improved LV contractility, despite maintained renal dysfunction Prevented the increase in cardiac α‐SMA expression, no effect on TGF‐β1 expression
Lachaux et al. (2018)	Zucker *fa\fa* rat, a model of metabolic syndrome cardiorenal injury	Finerenone	Short‐term: improvement in cardiac perfusion, reduced LV systolic diameter, decreased LV ROS production Long‐term: reduced cardiac hypertrophy, fibrosis and dysfunction
Michea et al. (2008)	Subtotal nephrectomy CKD model in rats	Spironolactone	Attenuated LV hypertrophy and prevented increased cardiomyocyte size in both ventricles, despite no effect on BP Attenuated LV oxidative stress
Clinical studies			
Boesby, Elung‐Jensen, Strandgaard, and Kamper (2013)	Stage 3–stage 4 CKD	Eplerenone	Attenuated pulse wave reflections (as measured by the Augmentation Index) after 24 weeks No effect on pulse wave velocity or ambulatory arterial stiffness index but may be underpowered and study period may have been too short
Charytan et al. (2019)	Haemodialysis patients	Spironolactone	No effect on echocardiographic parameters measured, although study was of an exploratory design
Edwards et al. (2010) and Edwards, Steeds, Stewart, Ferro, and Townend (2009)	“Early” CKD (stage 2–stage 3)	Spironolactone	Improved LV systolic and diastolic function, LV hypertrophy and arterial stiffness (pulse wave velocity\analysis, aortic distensibility)
Eschalier et al. (2013) (EMPHASIS‐HF)	Patients ≥ 55 years old with heart failure and reduced ejection fraction, including patients with mild or moderate CKD	Eplerenone	Reduced the risk of CV death or hospitalization for heart failure; as effective in CKD patients as in non‐CKD patients
Hammer et al. (2019)	Haemodialysis patients	Spironolactone	No change in LV mass or LV ejection fraction
Matsumoto et al. (2014)	Haemodialysis patients	Spironolactone	Reduced risk of cerebrovascular\CV death or hospitalization due to a cerebrovascular\CV event
Pitt et al. (2013) (ARTS)	Patients with heart failure and reduced left ventricular ejection fraction and mild to moderate CKD	Spironolactone vs. finerenone	Finerenone decreased the levels of B‐type natriuretic peptide, amino‐terminal pro‐B‐type natriuretic peptide to the same extent as spironolactone
Quach et al. (2016)	Meta‐analysis of 9 trials (829 patients, 2005–2015) in dialysis patients, with or without heart failure	Spironolactone and eplerenone	Decreased risk of CV mortality (relative risk 0.34) and all‐cause mortality relative to controls (relative risk 0.40), however quality of evidence deemed low
Sato et al. (2003)	DN	Spironolactone	Reduced LV mass index, without BP change
Taheri et al. (2009)	Haemodialysis in patients with moderate or severe heart failure	Spironolactone	Improved ejection fraction and LV mass compared with placebo

Abbreviations: BP, blood pressure; CKD, chronic kidney disease; CV, cardiovascular; DN, diabetic nephropathy; LV, left ventricular; TGF‐ß1, transforming growth factor‐ß1.

### 5.1 Possible adverse effects of MRAs

MRAs have the potential to reduce renal blood flow and GFR. Small decreases in eGFR are not infrequently reported in people receiving MRAs, likely reflecting reversal of hyperfiltration (Bolignano et al., 2014; Pisoni et al., 2012; Schjoedt et al., 2004). Although “worsening renal function” (based on eGFR) was described in large cardiovascular trials, mortality rates remained improved (Pitt et al., 1999, 2003; Zannad et al., 2011).

Hyperkalaemia is a concern with MRA treatment in human medicine, preventing their prescription in many instances (Maggioni et al., 2013). Individual studies report various effects on plasma potassium concentrations following MR blockade, including no difference between placebo and treatment (Epstein et al., 2006; Gonzales Monte et al., 2010; Sato et al., 2003) and increased hyperkalaemia incidence (Ando et al., 2014; Bianchi et al., 2006; Chrysostomou et al., 2006; Pisoni et al., 2012; Quach et al., 2016; Rachmani et al., 2004). Even though meta‐analyses conclude that MRAs (in addition to ACEIs and\or ARBs) increase the risk of hyperkalaemia, the mean increase in potassium levels with treatment is very small compared with placebo (0.26 mM) (Bolignano et al., 2014) and compared with baseline (0.19 mM) (Currie et al., 2016). Many trials excluded patients with high‐normal baseline circulating potassium concentrations, however. Even when statistically significant, increases in serum potassium are deemed “clinically modest,” and generally, the benefits of MR blockade are deemed greater than the risk of clinically relevant hyperkalaemia (Pisoni et al., 2012; Pitt et al., 1999, 2003).

Other adverse effects of spironolactone are related to its anti‐androgenic and progestogenic properties and include gynaecomastia, impotence, menstrual irregularities and mastalgia (Kolkhof & Borden, 2012; Matsumoto et al., 2014; Pitt et al., 1999; Ponda & Hostetter, 2006). These effects are not reported with eplerenone due to its increased MR selectivity (Ando et al., 2014; Pitt et al., 2003; Zannad et al., 2011) and would not be an issue in treating a cat population which are predominantly neutered.

## 6 ALDOSTERONE\MR ACTIVATION IN FELINE CKD

Understanding aldosterone's ability to promote renal injury in laboratory animals and humans provides a convincing basis for its potential role in feline CKD. There is limited information available regarding aldosterone\MR activation in this species. Although reference ranges for plasma aldosterone concentrations have been determined, the pulsatile nature of aldosterone release and effect of diet (sodium and potassium intake) may contribute to large intra‐ and interindividual variation (Buranakarl, Mathur, & Brown, 2004; Syme et al., 2007; Yu & Morris, 1998). Primary hyperaldosteronism, either due to adrenal gland neoplasia (Ash, Harvey, & Tasker, 2005) or due to hyperplasia (Javadi et al., 2005), is recognized in cats and, as in people, is associated with progressive renal disease and histopathological changes encompassing hyaline arteriosclerosis, glomerulosclerosis and tubulointerstitial fibrosis (Javadi et al., 2005).

As in laboratory species and human patients, RAAS activation is an important factor in the pathogenesis of feline CKD (Ames et al., 2019). Plasma renin, aldosterone, angiotensin I and angiotensin II are increased in cats with experimentally induced CKD following renal ischaemia\reperfusion injury (Watanabe & Mishina, 2007). Models employing renal wrapping exacerbate RAAS activation, resulting in more pronounced hypertension, proteinuria and histopathological changes (Buranakarl et al., 2004; Mathur et al., 2004). RAAS activation is further exacerbated by low sodium intake in this model (Buranakarl et al., 2004) and also occurs in cats with naturally occurring CKD which are transitioned onto (relatively sodium‐restricted) renal diets (Syme, 2003). Although experimental data support RAAS activation in feline CKD, it may not directly translate to naturally occurring disease, as plasma renin activity and aldosterone concentrations do not differ between normotensive azotaemic CKD cats and nonazotaemic age‐matched controls (Jepson et al., 2014). Mishina et al. (1998) reported increased circulating renin, angiotensin II and aldosterone levels along with increased blood pressure in cats with CKD, although it is unclear whether the groups were age‐matched. As in people and rodents, local (intrarenal) RAAS is likely of importance; three studies to date have investigated this using immunohistochemistry in naturally occurring feline CKD (Mitani, Yabuki, Sawa, Chang, & Yamato, 2013; Mitani, Yabuki, Taniguchi, & Yamato, 2013; Taugner, Baatz, & Nobiling, 1996). Renin expression was not associated with azotaemia severity or histopathological lesions (Taugner et al., 1996). Tubular and interstitial angiotensin II, but not ACE or ACE2 expression, was correlated with glomerulosclerosis and tubulointerstitial inflammation (Mitani, Yabuki, Sawa, et al., 2013; Mitani, Yabuki, Taniguchi, et al., 2013). Intrarenal aldosterone has not been examined, although assessment of renal 11β‐HSD activity has been attempted by urinary cortisol–cortisone ratio measurement; cats with CKD had a lower ratio, not supportive of the hypothesis that decreased excretion of active glucocorticoid may potentially reflect excessive MR stimulation in this population (Walker, Elliott, & Syme, 2009).

Aldosterone appears to be associated with feline systemic hypertension, a common finding in cats with CKD. Plasma aldosterone levels are higher in hypertensive azotaemic cats than nonhypertensive cats with and without renal disease (Jensen et al., 1997; Jepson et al., 2014). Lower plasma potassium tends to be a risk factor for feline hypertension in epidemiological studies, providing support for MR activation (Jepson et al., 2009; Sansom, Rogers, & Wood, 2004; Syme, Barber, Markwell, & Elliott, 2002), although blood pressure is not directly associated with plasma or urinary aldosterone concentrations (Syme, Barber, et al., 2002; Syme et al., 2007; Williams et al., 2013). Increased plasma aldosterone concentration is not seemingly driven by plasma renin activity, as cats with concurrent CKD and hypertension have variable or decreased renin compared with controls, resulting in increased aldosterone‐to‐renin ratios (Jensen et al., 1997; Jepson et al., 2014; Syme, Markwell, et al., 2002). Given that increased circulating aldosterone in cats with concurrent CKD and hypertension does not appear to be secondary to increased renin or hyperkalaemia, alternative explanatory mechanisms include primary adrenal‐dependent pathology, local MR activation, altered sensitivity to stimuli which dictate aldosterone release or reduced aldosterone degradation (Buranakarl et al., 2004).

### 6.1 MRA use in cats

The optimal way to inhibit RAAS activation in feline CKD has yet to be determined, and to date, treatment has consisted of ACEI and\or ARB therapy. In many countries, the ACEI benazepril is licensed for treating proteinuria associated with CKD in cats and the ARB, telmisartan, is licensed for feline hypertension and proteinuria treatment (Coleman et al., 2019; Glaus, Elliott, Herberich, Zimmering, & Albrecht, 2019). Benazepril ameliorates glomerular capillary hypertension, increases GFR and reduces proteinuria in a partial renal ablation model (Brown et al., 2001), and reduces proteinuria in naturally occurring CKD (King, Gunn‐Moore, Tasker, Gleadhill, & Strehlau, 2006; Watanabe & Mishina, 2007). Telmisartan is as efficacious as benazepril in reducing urine protein\creatinine ratio in clinical cases (Sent, Gössl, Elliott, Syme, & Zimmering, 2015). Although ACEIs and ARBs successfully reduce proteinuria, a factor associated with reduced survival (King et al., 2007; Kuwahara et al., 2006; Syme et al., 2006), the present studies investigating these drugs in feline CKD have important limitations (e.g. are underpowered or not designed to test long‐term outcomes) which prevent definitive conclusions from being made about their effect on CKD progression and prognosis in cats (King et al., 2006; Sent et al., 2015; Watanabe & Mishina, 2007). Aldosterone breakthrough has not been studied in cats with CKD receiving long‐term ACEIs or ARBs.

Two studies have investigated spironolactone in feline cardiac disease. Relevant to CKD pathology, feline hypertrophic cardiomyopathy is characterized by significant interstitial fibrosis and arteriosclerosis (Fox, 2003). In a small study of hypertrophic cardiomyopathy in Maine Coons, four of 13 treated cats developed severe ulcerative facial dermatitis approximately 2.5 months into treatment which the authors attributed to spironolactone (MacDonald & Kittleson, 2008). The dosage used in this study (2 mg\kg twice daily) was twice the recommended dosage in dogs (Guyonnet, Elliott, & Kaltsatos, 2010), and feline herpesvirus was not sufficiently ruled out as a possible cause. One cat also developed myelodysplasia. Cutaneous drug reactions are sporadically reported in people receiving spironolactone (Gupta, Knowles, & Shear, 1994) and spironolactone‐induced agranulocytosis, and aplastic anaemia is also recognized (Ibáñez, Vidal, Ballarín, & Laporte, 2005). A second study reported no dermatological adverse effects of spironolactone (1.7–3.3 mg\kg once daily) over a 15‐month treatment period, and the prevalence of adverse events was similar between the treatment and placebo groups (James et al., 2018). The risk of hyperkalaemia with ACEI and spironolactone co‐therapy is emphasized in veterinary medicine, although combination therapy appears well‐tolerated in cats and dogs with heart failure (James et al., 2018; Lefebvre et al., 2013). Cats with mild–moderate CKD tend to have lower than normal plasma potassium concentrations, with a 12%–20% prevalence of hypokalaemia (Elliott & Barber, 1998; King et al., 2007; Ross et al., 2006). MRA therapy reduces hypokalaemia risk in people (Pisoni et al., 2012; Pitt et al., 2003), a potentially beneficial effect in feline patients.

## 7 CONCLUSIONS

Given the expanding evidence base from in vitro and in vivo experimental studies and from human medicine, it seems likely that aldosterone and MR activation is an important player in the pathogenesis of feline CKD. It must be noted, however, that experimental models may not be directly translatable to the clinical situation and that differences in CKD pathogenesis exist between humans and cats. Furthermore, there is a need for human studies evaluating the effect of MRAs on mortality and CKD progression as primary end points. MR blockade may protect the kidney from ischaemia, repeated bouts of which may be responsible for the loss of functioning renal tissue in the cat. Secondly, MRAs reduce proteinuria in other species, and as proteinuria is associated with renal fibrosis and disease progression in the cat, therapy may have beneficial effects on survival. Additionally, MR activation appears to contribute to hypertension in cats with CKD, and MRAs may reduce blood pressure and the subsequent risk of proteinuria and further ischaemic renal damage. Finally, disturbances in mineral and bone metabolism occur in feline CKD and MR blockade may prove beneficial in reducing secondary vascular and soft tissue mineralization, as has been shown experimentally. Field studies investigating aldosterone breakthrough and the use of MRAs in naturally occurring feline CKD, where the goal remains to slow disease progression, are indicated.

## CONFLICT OF INTEREST

JE is a member of the International Renal Interest Society, which is sponsored by Elanco Animal Health Ltd. JE has acted as a paid consultant for CEVA Animal Health, Boehringer Ingelheim Ltd., Kindred Bio Inc., Orion Ltd., Royal Canin Ltd., Idexx Laboratories Ltd. and Waltham Ltd. JE is in receipt of grant funding from Royal Canin Ltd., Elanco Animal Health Ltd. and Idexx Laboratories Ltd. None of the authors has any other financial or personal relationships that could inappropriately influence or bias the content of the paper. CEVA Animal Health, who market spironolactone for the treatment of canine congestive heart failure caused by valvular regurgitation, played no role in the preparation of this manuscript.

## AUTHOR CONTRIBUTION

SS, CWJ and JE were responsible for the writing of this manuscript and have read and approved the final manuscript.
